# Decision Tree Pattern Recognition Model for Radio Frequency Interference Suppression in NQR Experiments

**DOI:** 10.3390/s19143153

**Published:** 2019-07-17

**Authors:** Mona Ibrahim, Dan J. Parrish, Tim W. C. Brown, Peter J. McDonald

**Affiliations:** 1 Department of Physics, University of Surrey, Guildford GU2 7XH, UK; 2 Institute for Communication Systems, University of Surrey, Guildford GU2 7XH, UK

**Keywords:** nuclear magnetic resonance, nuclear quadrupole resonance, radio frequency interference, machine learning classification

## Abstract

Radio frequency interference places a major limitation on the in-situ use of unshielded nuclear quadrupole or nuclear magnetic resonance methods in industrial environments for quality control and assurance applications. In this work, we take the detection of contraband in an airport security-type application that is subject to burst mode radio frequency interference as a test case. We show that a machine learning decision tree model is ideally suited to the automated identification of interference bursts, and can be used in support of automated interference suppression algorithms. The usefulness of the data processed additionally by the new algorithm compared to traditional processing is shown in a receiver operating characteristic (ROC) analysis of a validation trial designed to mimic a security contraband detection application. The results show a highly significant increase in the area under the ROC curve from 0.580 to 0.906 for the proper identification of recovered data distorted by interfering bursts.

## 1. Introduction

Nuclear magnetic resonance (NMR) and nuclear quadrupole resonance (NQR) are widely used as laboratory techniques to characterize materials and processes. However, there is also interest in using these methods for in-situ sensing applications, with a growing list of established and nascent application areas—the most developed being oil-well logging [1]. Other areas include in- or by-line quality control and assurance in food processing, agri-sciences, the built environment, and security [2,3]. In these applications, measurements are typically made at a low frequency (1–10 MHz), where the sensitivity and signal-to-noise ratio are major practical issues. Noise, in its broadest sense, can be incoherent, such as thermal white noise emanating from the NMR/NQR sensor coil, or coherent interference. Incoherent noise is removed by signal averaging or, in-extremis, the use of specialist hardware based on, for instance, superconducting quantum interference device sensors [4]. Coherent noise includes internal acoustic ringing of the apparatus following an excitation pulse, and also external radio frequency interference (RFI) [5]. Normally, acoustic ringing is eliminated using an excitation pulse phase cycling scheme, but where this is not sufficient, additional methods have been proposed, for instance, active and passive Q-damping and reverse-phase pulses [6]. RFI, where possible, should be eliminated using Faraday shielding. In the case of oil-well logging, the Earth provides a natural Faraday shield. However, in other instances, such as security and tree moisture applications, shielding is limited or impossible.

When RFI is detected in unshielded NMR or NQR experiments, it often saturates the receiver and generally completely obscures the signal. Signal averaging is not possible, as RFI occurs with sufficient frequency to cumulatively obliterate the entire time domain signal in the averaged data. Therefore, it needs to be removed. The experimental bandwidth coupled with the spread of different carrier frequencies in the RFI is such that direct bandpass filtering is not workable. Therefore, an alternate signal processing approach is required.

In modern digital spectrometers, NMR or NQR data processing can be thought of as comprising of two principle steps. The first is the demodulation and filtering of the radio frequency signal to reveal the baseband signal in the time domain. The second is the processing of that signal either in the time or reciprocal frequency domain, to yield chemical specific or microstructural specific information about the sample, which may be very rich in content. For security applications, such as contraband detection, NQR is often favored, and the second step may be as simple as to identify if an NQR signal is, or is not, present.

There have been previous attempts going back many years, in both hardware and software, to remove the coherent interference from NMR/NQR signals. In the literature [7], tests of landmine detection are reported, which include running multiple RFI sensors in parallel with the principal NQR experimental sensor. The data from the additional sensors is used to estimate the RFI at the site of the principal sensor, and hence subtract it out from the NQR data. No specific figures for the RFI removal efficiency are given. However, resultant from the tests, a false alarm rate of circa 0.1 mine per square meter explored was reported with “100%” mine detection efficiency. Further multiple channel approaches for RFI mitigation in NQR applications can be found in the literature [8,9,10]. All of these studies show good results. For instance, in static trials in the literature [8] (the detector moved across the field in [7]), interference suppression by 54%was achieved.

Advanced filtering methods have also been studied. In the literature [11], a single sensor least mean squares technique was used to estimate the background interference, and a Bayesian discriminant algorithm was used to recover the NQR signal from the remaining data. The same group used Kalman filtering in the literature [12]. The results are compared to a static peak energy detector. The Bayesian approach typically (using multiple data sets) improves the reverse diagonal detection performance/false alarm rate on a receiver operating characteristics curve for effectiveness trials from 75%/25% to better than 85%/15%. In the literature [13,14] a method is proposed to remove the RFI from both the stochastic and conventional NQR data using a signal of interest free data. For the simulated data with a signal to noise ratio of typically −30 dB corrupted by RFI at up to 100 dB, detection probabilities in excess of 80% (conventional) and 90% (stochastic) are reported. Although this method shows good results for improving the signal-to-noise ratio (SNR), the authors assume that the RFI lies in a low-rank linear interference subspace. Such an RFI is less likely to mimic real world RFI that is, in most cases, nonlinear and non-stationary. More recently, an advanced filtering method, dubbed interference-cancelled echo-train approximate maximum-likelihood, has been introduced by some of the same authors [15]. This is specifically designed to target RFI, with a significant fraction of spectral width very close to, but not on, the NQR frequency, which is also non-stationary. Comparably good probability of detection/false alarm rates are reported from simulated data for this harder problem. The results for the laboratory acquired experimental data are strikingly better (>95%/5%), although it is not evident that the RFI is as challenging. In the literature [16], the authors also allow for non-stationary RFI. The algorithms perform well, with more than a 90% detection probability for signal to interference ratios, up to of the order of 40 dB. In the literature [17], a singular value decomposition (SVD) algorithm is introduced for RFI cancellation in NMR signals. By removing the singular values corresponding to the RFI and noise, the amplitudes of the interference peaks are suppressed by 10 dB. Finally, the authors of [18] suggested a method based on the linear predictive coding of RFI through NMR signals. This method is especially applicable to measurements in highly inhomogeneous magnetic fields, where the echo duration is extremely short.

In this paper, we explore the opportunity to use machine learning to reduce the impact of RFI on NQR data. Furthermore, unlike most of the earlier studies that advanced signal processing methodologies, we specifically address burst-mode RFI that, as discussed below, is typical of aeronautical mobile communications interference rather than quasi-continuous RFI typical of, for example, radio transmissions. We have previously shown that burst-mode RFI dominates in our laboratories.

Machine learning could be invoked for both parts of the processing; first, the identification and removal of RFI, and second, information retrieval from the demodulated signal. However, as the analysis of the time domain demodulated signal is application-specific, whilst the need to remove RFI is generic, we focus on the first—the identification and removal of RFI. Specifically, we develop, train, and apply a decision tree model to identify instances of burst-mode RFI. A very simple strategy to remove this RFI is then tested. We remove the RFI affected parts of the data and replace it with data from a repeat, but differently affected, experiment in a weighted averaging process. In order to measure the improvement that machine learning brings to the data processing, we use a trial involving human volunteers to measure the reliability with which they can “see” a signal in the processed data. This (ideally) yes/no criterion is typical of the first stage of security applications, such as baggage scanning at airports. While we address a security type application, the generic nature of the RFI identification and removal makes the methodology widely applicable.

Two final points are noted here—the first is that while machine learning has advanced at a great pace with new protocols continuing to be proposed, we have found it sufficient to use a well-established method, decision tree classification, for our purposes. As stated below, it has been proven to be more successful than the alternatives that we have assessed. The second is that, to our knowledge, there is no work to explore machine learning for RFI suppression in NMR/NQR applications specifically. However, it is being used to explore RFI suppression in other areas of technology [19].

Section 2 covers a brief description of the experimental setup, and details the emulated RFI within a Faraday shield. Section 3 details the suggested RFI suppression technique, it includes a description of the decision tree model and how it is going to be used for RFI suppression. Section 4 covers the results together with the validation and some discussion, and a conclusion in Section 5 ends the article.

## 2. Experimental Setup

### 2.1. Radio-Frequency Interference: Recording and Reproduction

**Figure 1:**
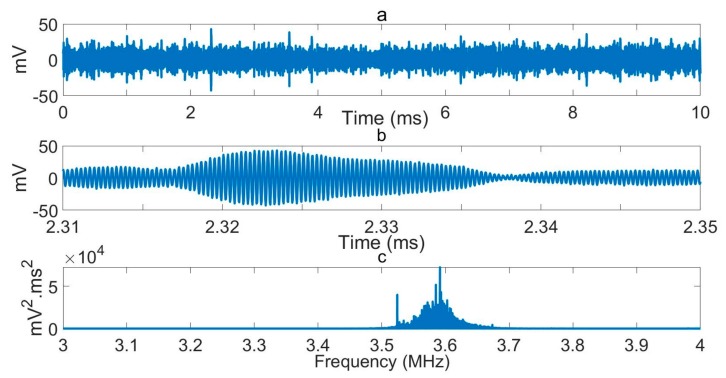
Exemplar radio frequency interference (RFI) recorded through a tuned nuclear quadrupole resonance (NQR) coil and bandpass preamplifiers, as used in the experiments, save that the coil is unshielded. A series of short duration noise bursts are seen with carrier frequencies spread across the NQR frequency bandwidth. (**a**,**b**) signal elements at different time resolutions. (**c**) Fourier transform of a 100 ms data sample. The data were recorded at 31 MHz with a 10 MHz analogue low pass filter in the line.

We previously recorded a significant volume of RFI within the environs of our laboratories at frequencies in the range of approximately 0 to 10 MHz [18]. Outdoor measurements were undertaken at our laboratories, where it was found that a significant fraction of the RFI comprised pulsed aircraft communication data. We are located close to two major London international airports. Repeat measurements have been made recently, three years later. They confirm the long-term significance of the problem. Figure 1 shows a recent short time domain exemplar RFI signal and its Fourier Transform in the frequency window of interest, so as to illustrate the nature of the signal to be tackled. The signal was recorded directly using the unshielded NQR detector used in this work. Numerous interfering carrier and sideband channels can be observed.

In order to have a reproducible, controlled, RFI of a variable amplitude, frequency, and phase, the recorded RFI data were given a binary threshold for “on” or “off”. The resultant mask was used to switch controlled carrier and sideband signals at frequencies around the NQR frequency with a desired amplitude and phase to high and low states. Hence, we can generate a known, but randomized, pseudo-RFI that mimics real-world burst-mode RFI.

### 2.2. Experimental Testbed

**Figure 2:**
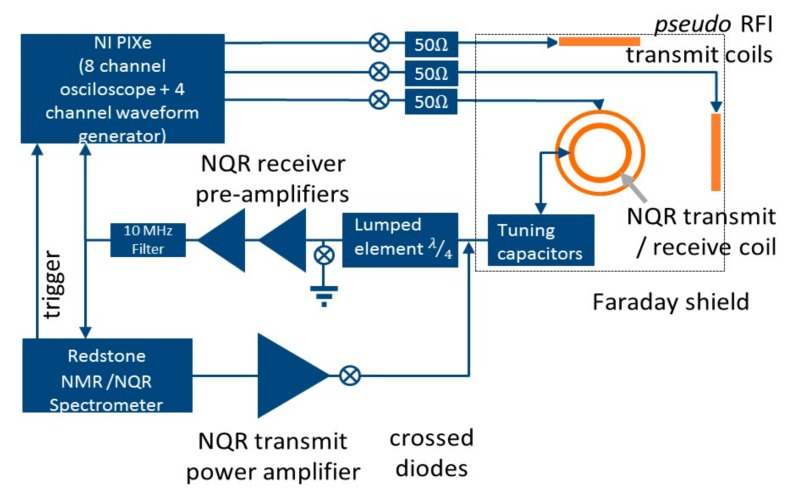
A schematic diagram of the experimental testbed.

Although our target application is in-situ NQR, progress is best made on a test bed within a Faraday shield, into which the pseudo-RFI is deliberately broadcast. To that end, a Faraday shielded demonstrator was built within a 70 × 70 × 70 cm^3^ box. Figure 2 is a schematic of the demonstrator. The demonstrator contained the NQR spectrometer coil and three further coils to transmit RFI. Each coil was cylindrical, circa 14 cm long and 7.8 cm in diameter, with an internal volume of 670 cm^3^. The coils comprised of 11 conductor turns. The NQR coil was parallel-series tuned to 3.6 MHz and 50 Ω, with circa 110 pF and 50 pF capacitors. They had a nominal bandwidth of 0.2 MHz. The NQR coil was used to contain the sample, to transmit radio-frequency NQR excitation pulses, and to detect NQR signals, as well as detect RFI. The NQR coil was mounted centrally on the demonstrator base plate. The RFI transmission coils were mounted on the top plate and two side walls, and oriented in orthogonal directions. These enabled the simultaneous broadcasting of three RFI channels representing three real sources. The three channels of pseudo RFI were broadcast into the box using two PXIe 5423 dual channel (four channels maximum) arbitrary waveform generator cards run by a PXIe-8840 computer, all contained in a PXIe 1071 four bay chassis. The cards transmitted the digitized RFI at 10 MHz. The PXIe system was programmed in LabVIEW.

The NQR excitation pulses were generated by a Redstone spectrometer operating at 3.6 MHz, amplified and broadcast to the NQR coil. Signals from the NQR coil could be detected and processed by the Redstone spectrometer in the normal way, enabling the functioning of the base experiment to be tested. More usually, the signal from the NQR coil was recorded by a PXIe 5170R eight Channel Oscilloscope. The data were continuously recorded throughout the experiment at an oversampled rate of 10 MHz.

### 2.3. NQR Carr Purcell Meiboom Gill (CPMG) and Free Induction Decay (FID) Experiments

Sodium Nitrite has three nitrogen NQR resonances at circa 1.0, 3.6, and 4.6 MHz at room temperature. The resonance at 3.6 MHz offers a relatively safe (in the health and safety context) analogue for a wide range of contraband materials.

With all of the cables and coils connected to the equipment, and with the Faraday shield in place, but with no deliberately broadcast RFI, an ^14^N free induction decay (FID) experiment using a 0.2 kg sodium nitrite as a sample easily achieved a signal to noise ratio (SNR) of 140 in 32 repetition scans. Without the shielding, the same experiment produced an SNR of 2.2. The $P_{90}$ excitation pulse length was 240 µs.

More generally, Carr Purcell Meiboom Gill (CPMG) experiments were performed. The sodium nitrite sample mass was varied between 0 and 200 g in steps of 50 g. As the resonance frequency can be strongly temperature dependent (typically few kHz/K), and in a real-world application the sample temperature may be, at best, poorly specified, the NQR frequency was varied randomly according to a Gaussian distribution, with central frequency 3.6 MHz and standard deviation 0.0065 MHz, between the experiments. The NQR coil was not retuned with changing the NQR frequency.

All of the other key NQR parameters were also varied, including the following: the number of echoes, $\;n_{echo}\in \left\{4,8,12\right\}$; the echo spacing, 500<τ<3300 μ s (linear distribution); and the excitation pulse length, $186<P_{90}<293$ μ s (linear distribution, with $P_{90}^{true}=240$ µs). The refocusing pulse length was always twice the excitation pulse length. For each selected parameter set, a “set” of 64 echo train “scans” were individually recorded.

Three channels of different pseudo random RFIs were broadcast into the experiments simultaneously. For each channel and for every individual scan, the RFI mask, amplitude (*A*), carrier and sideband frequencies $\left(f_{c},f_{s}\right)$, and phase $\left(\varphi \right)$ were randomly varied in and around the bandwidth of the NQR experiment. The lowest RFI amplitude (excluding zero) was chosen so that, with an RFI carrier frequency of 3.6 MHz, a signal was detected in the NMR coil with an amplitude comparable to the smallest sodium nitrite sample signal. The largest amplitude was chosen so that it saturated the spectrometer receiver and was perceived as an approximate square-wave. The RFI mask and other parameters were recorded so as to enable the subsequent reconstruction of the RFI, if, and when, desired, for example, for the training the decision tree model.

Three series of data were recoded spanning the full range of parameter space, namely:

1. Data Series I: Identical NQR parameters were run for each of the five sample masses. A total of 120 data sets (equivalent to 7680 scans, equivalent to circa 51,200 echoes) were recorded. This first data set was used primarily for training the analysis algorithms. The data were recorded by one of us and made available—with all of the random acquisition parameters—to another who performed the training. Twenty percent of the data were recorded without RFI.
2. Data Series II: This was similar to Series I, in that repeated combinations of parameters were used for recording the data for each sample mass, however, the series contained only 40 sets (2560 scans and 24,576 echoes). The data and selected parameters ($P_{90}$, τ, number of echoes, and $f_{NQR}$), as would normally be known, were made available to the analyst. No information regarding the RFI or sample mass were available for the analyst.
3. Data Series III: This much larger volume of data contained 200 sets (12,800 scans and 102,400 echoes). The sample mass was now also varied randomly across the data sets. Only the raw data was made available to the analyst—in particular, no information was provided about the sample mass or RFI mask prior to the analysis.

## 3. Decision Tree Model for RFI Suppression

### 3.1. RFI Suppression by Decision Tree Classifier

**Figure 3:**
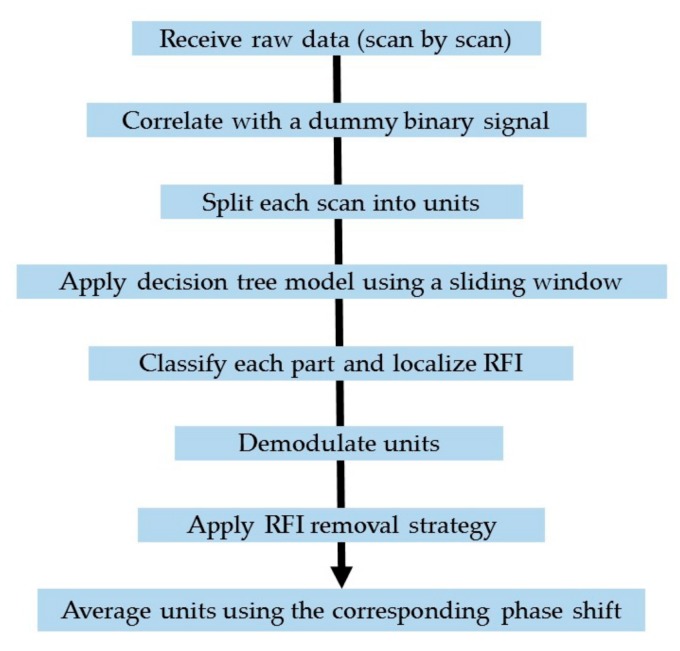
A flowchart of the RFI suppression using the decision tree model.

In this work, the decision was taken to perform this operation in three steps. First, we used a decision tree model to identify the locations of the RFI within the raw time domain signal before demodulation. Second, we demodulated the entire signal (including RFI). Third, we acted on the demodulated signal to suppress the RFI at the now known locations. These three actions are discussed separately and are shown in the flowchart in Figure 3.

Decision tree models are one of the most popular machine learning models because of their simplicity in term of the mathematical models used during training and predicting data, and because of their rapidity.

A decision tree classification is a machine-learning model based on sequentially partitioning the data into decreasingly small sets. The tree starts at a root node, where the data is divided into two smaller sets based on a binary “feature” test of the data—that is, upon whether some quantitative measure derived from the data is, for instance, greater or less than a threshold value. Features can be temporal- or frequency-based, or based on some characteristic of the probability distribution of the data [20,21,22,23]. Further successive binary splittings of the data are made, based on further feature decisions. At each splitting, a smaller set of data is formed, and the process continues until no more splitting is possible or the chosen maximum number of splits is achieved. At this point, the data are grouped into “classes”. A tree thereby comprises of nodes (decision points), branches (decision outcomes), and leaves (class or classification outcomes). The training program is designed to optimize the tree for classifying a particular type of data, given the known classes of the training data set. However, a fully-grown tree is likely to overfit the data, leading to poor accuracy on unseen data. Pruning is used to overcome overfitting. A discovered decision tree specific to this work is given in the results section to illustrate the process. Further discussions of decision trees can be found in the literature [24].

**Figure 4:**
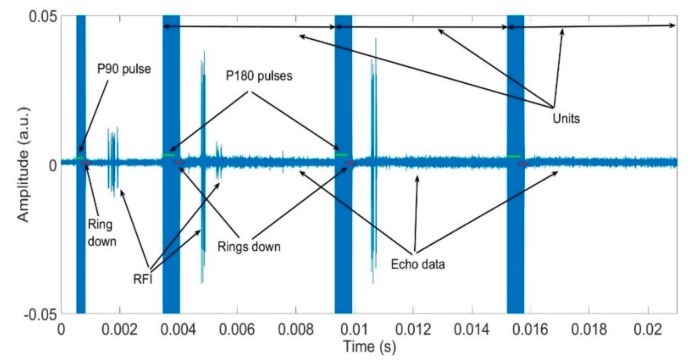
An exemplar recorded scan comprised of 3 units to illustrate the different component classes.

The data consists of NQR scans; these are time series (see Figure 4). Each scan comprises a number of “units”, defined as the time series from the start of one refocusing excitation pulse to the next. Each unit is composed of four main parts, namely: NQR excitation pulse, receiver ring down following saturation of the receiver by the pulse, echo, and RFI, as identified in the figure. The core idea of this work is to suppress the RFI from all units. Therefore, for the decision tree model, the four classes are the following: pulse, ring down, echo, and RFI. Note that the data starts with a pulse half the length ($P_{90}$) of all of the others ($P_{180}$), and that this is followed by an interval half the duration of all of the others. This is standard for the experiment; the useful data is in the wider intervals.

**Figure 5:**
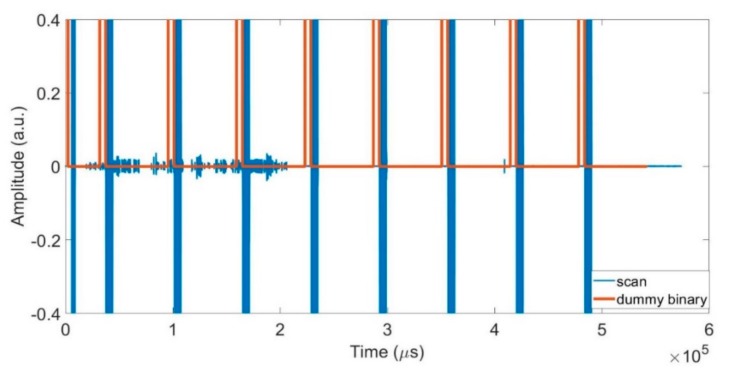
A single recorded data scan (in blue) comprised of 8 units with the corresponding dummy binary signal (in orange).

Training data were prepared using recorded scans from the first experimental series. Each scan was separated into units, each comprising (ideally) an excitation pulse at the start, followed by echo data. In practice, because the PXIe and Redstone instruments ran asynchronously, there was a variable delay at the start of each recorded scan. In order to eliminate this delay and ensure accurate data timing between the scans and units, a dummy binary signal (x) was created, in such a way that it was equal to zero all over the scan, save that it is equal to one at the expected location of the pulses. The dummy signal started with $P_{90}$; it did not include the varying gap before the first pulse in each scan, as shown in Figure 5.

The delay between the dummy binary and an actual scan, y, is calculated using the normalized cross-correlation between the two signals, at all possible lags, as follows:

(1) $$\;R_{xy}\left(m\right)=E\left\{x_{n+m}y_{n}^{*}\right\}=E\left\{x_{n}y_{n-m}^{*}\right\}$$

The calculated delay is given by the negative of the lag, for which the normalized cross-correlation has the largest absolute value. This delay, and the known pulse gap (τ) enabled different scans to be accurately aligned and divided into units.

For model training, it is required to characterize each region of every training scan as one of the following four classes: pulse, ring down, echo data, or RFI. As the number of units is large, this process was automated. Identification of the $P_{180}$ pulse is straightforward, as it occurs at the start of the unit and has a known duration. Furthermore, for training, RFI is identified using the RFI transmit mask previously discussed. In order to separate the ring down and therefore find the beginning of the echo within a data unit, we run a moving average filter on the data region immediately following the $P_{180}$ pulse. The ring down is judged to have ended where the modulus of the data intensity, $\left|y\right|$, first equals the time averaged root mean square plus one standard deviation of the remaining echo signal. The echo data is identified as all that remains after the separation of the pulse, ring down, and RFI. The training data is thus fully separated into classes.

For each of these parts, we passed a sliding window of 20 µs (about 218 data points) across the data—the windows did not overlap. For each of these windows, a set of 27 features were computed, ready to be input into the decision tree classifier. The features comprised a combination of temporal and frequency characteristics of each data window, as well as statistical characteristics. The chosen features were documented as follows.

For each training 20 µs data segment (y) and its magnitude ($\left|y\right|$), we measured the following features: the statistical means ($\bar{y}$) and ($\bar{\;\left|y\right|}$), the standard deviations, as follows:

(2) $$\;\;\;\;\sigma =\sqrt{\frac{\sum_{i=1}^{N}\left(y_{i}-\bar{y}\right)}{N}}$$

and

(3) $$\sigma_{\left|\right|}=\sqrt{\frac{\sum_{i=1}^{N}(|y_{i}|-\bar{\left|y\right|})}{N}}\;$$

where N is the unit length, the kurtosis, as follows:

(4) $$\;k=\frac{\sum_{i=1}^{N}{\left(y_{i}-\bar{y}\right)}^{4}/N}{\sigma^{4}}$$

and

(5) $$k_{\left|\right|}=\frac{\sum_{i=1}^{N}{\left(|y_{i}|-\bar{\left|y\right|}\right)}^{4}/N}{\sigma_{\left|\right|}{}^{4}}$$

the skewness

(6) $$sk=\frac{\sum_{i=1}^{N}{\left(y_{i}-\bar{y}\right)}^{3}/N}{\sigma^{3}}\;$$

and

(7) $$sk_{\left|\right|}=\frac{\sum_{i=1}^{N}{\left(|y_{i}|-\bar{\left|y\right|}\right)}^{3}/N}{\sigma_{\left|\right|}{}^{3}}$$

and the 25th and 75th percentile, and the 25th and 75th cumulated sums for both y and $\left|y\right|$. For each data unit, we measured the average of the derivative ($\bar{\;y^{’}}$), the power

(8) $$\frac{\sum_{i=0}^{N-1}y_{i}{}^{2}}{N},$$

the envelope, and the start and end times of the segment.

The frequency features are as follows: the maximal frequency and its height, the number of peaks, the sum of the heights of these peaks, the average distance that separates two neighboring peaks, and the area under the curve in the spectral density graph.

**Figure 6:**
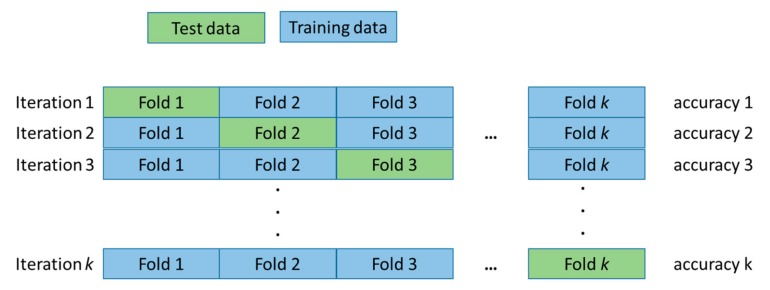
An illustration of the *k*-fold validation method.

In this work, we used a supervised decision tree model, as the data and the classes were known. Hence, at the training stage, the inputs of the model were the features, and the target was the corresponding classes. Labelled training data was fed to the decision tree model. We set 100 as the maximum number of splittings for the model, and adopted a *k* = 25-fold cross validation methodology. The *k*-fold cross validation consisted of portioning the available data into *k* bins (or folds) of equal size. One of these subsets was picked as a test set. The remaining *k* − 1 sets were put together into a training set, so as to train the machine learning algorithm and find the accuracy on the test set. The model was run *k* times, with each set used in turn as the test set, in order to find the general accuracy of the model by averaging across the *k* runs. The *k*-fold cross validation was illustrated in Figure 6.

The data training was performed in the MATLAB (data pre-processing, training algorithms, and validation) and Python (training decision tree model and feature importance) programming languages.

### 3.2. Demodulation

The data were quadrature demodulated in the normal fashion, using standard MATLAB^®^ functions. A low pass filter of 10 kHz was applied. Checks were made that data recorded with the PXIe and demodulated in MATLAB were essentially the same as that demodulated by the spectrometer. An exact equivalence was not obtained, as the spectrometer filter detail was unknown.

### 3.3. RFI Suppression

**Figure 7:**
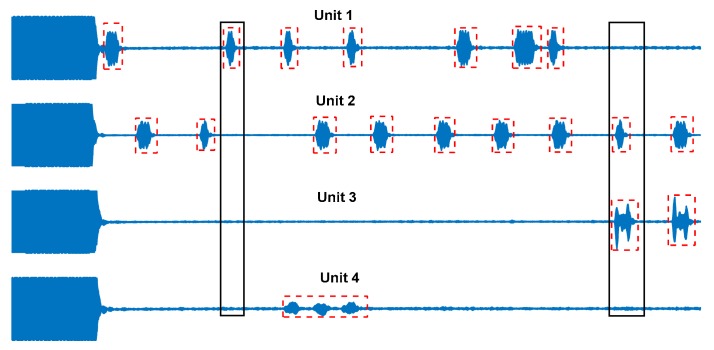
An illustration of RFI direct removal. The figure shows, from top to bottom, four data units, each comprising an excitation pulse and ring down at the start, echo data close to the baseline noise level, and multiple bursts of RFI. The instances of RFI are identified by the small dash-line red-rectangles. The two tall black rectangles highlight two segments of data differently affected by RFI. In the left-hand case, only Unit 1 is affected, and so the data from Units 2, 3, and 4 is averaged; in the right hand case, both Units 2 and 3 are affected, so Units 1 and 4 are used.

The RFI removal strategy chosen was the most straightforward possible. As the RFI was in burst mode, we did not need to adopt the complex filtering methodologies as needed more widely by others. A typical data set contained 64 scans. The methodology consisted of emptying the regions of data within individual scans that were classified by the decision tree model as containing RFI. At every time point, the data from the remaining scans that do not include RFI at that point is averaged. We term this “direct removal”, as illustrated in Figure 7.

## 4. Results of RFI Removal

### 4.1. Training

**Figure 8:**
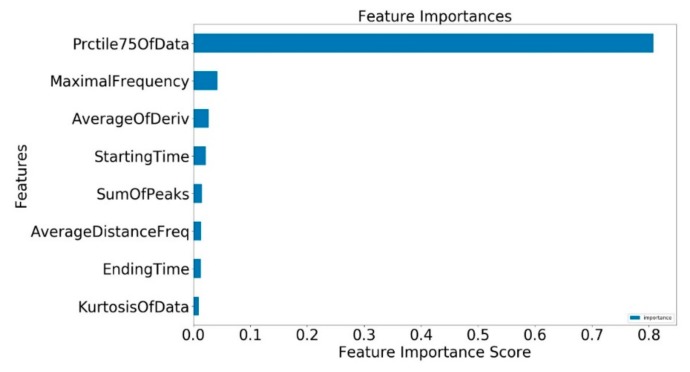
Feature importance scores of the eight most important features.

Training was first performed using all 27 features. Using the Scikit-Learn library in Python. We found that for each 20 μ s long data segment, the features that were most the important in determining whether it contained pulse, ring down, echo signal, or RFI were the following: the 75th percentile, the maximal frequency, the derivative average, the starting time of the segment within the whole data segment, the sum of the frequency peaks, the average distance between peaks, the end time, and the data kurtosis (as shown in Figure 8). On this basis, the training was repeated using just the most important eight, and then four, features.

**Figure 9:**
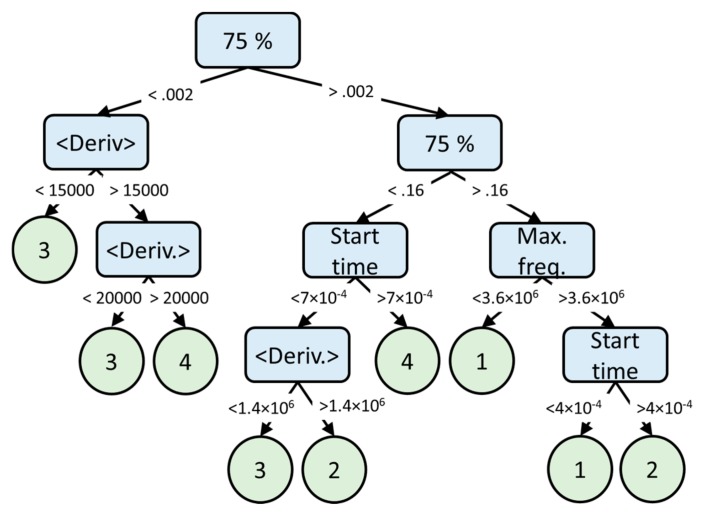
The training output, but very heavily pruned, decision tree based on the four most significant features. Nodes are shown light blue, leaves light green, and branches with numerical test conditions as arrows. The classes are as follows: (1) pulse, (2) ring down (3) echo data, and (4) RFI.

The topmost levels of the very heavily pruned, four-feature decision tree discovered by the training is shown in Figure 9 for illustrative purposes. Only eight nodes were retained. The full trees from 27, 8, and 4 features are available from the University of Surrey data repository.

**Figure 10:**
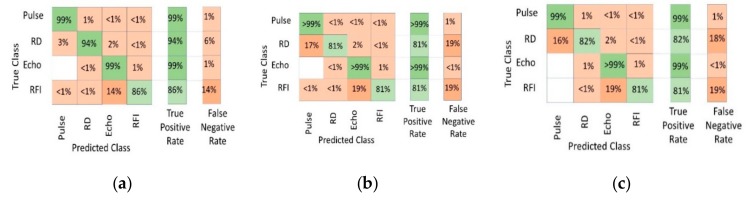
Confusion matrices of the decision tree model using the following: (**a**) 27 features, (**b**) 8 features, and (**c**) 4 features.

The confusion matrix of the trained RFI identification model for the tree based on all 27 features is shown in Figure 10a. An overall accuracy of 98.2% ± 0.2% was obtained on the training data. Specifically, the model correctly identified 86.0% ± 0.2% of the RFI occurrences. The false negative rate was therefore 14%. The machine got confused on 14% between echo and RFI, predominantly when the RFI amplitude was very close to the echo amplitude and when the modulation frequency was very close to the NQR frequency.

**Figure 11:**
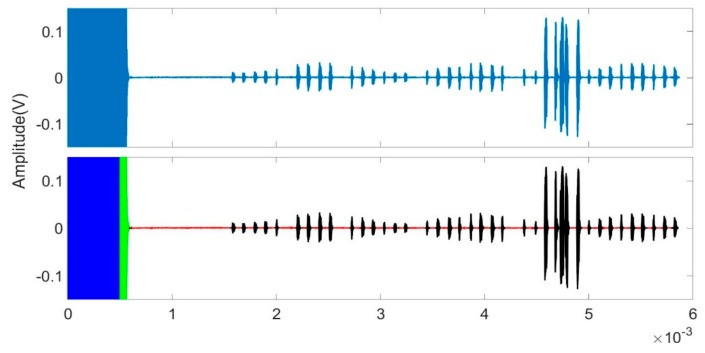
Recognition of the different parts of a random unit by the decision tree RFI suppression model. The original data is in the upper part of the figure. The lower part shows the classification result, where the blue, red, black, and green coloration represent the predicted pulse, echo, RFI, and ring down, respectively.

An example of how well the proposed model is able to correctly classify the different parts of a unit is shown in Figure 11. This figure shows that the proposed model can successfully identify all of the different parts of the unit. The resultant confusion matrices are shown for trees with fewer features (as shown in Figure 10b,c). Fewer features produce almost as good results. Notwithstanding the identification of ring down, the echo data and RFI were degraded, and so we continued to use 27 features throughout the remainder of this work.

### 4.2. Comparative Tests

**Figure 12:**
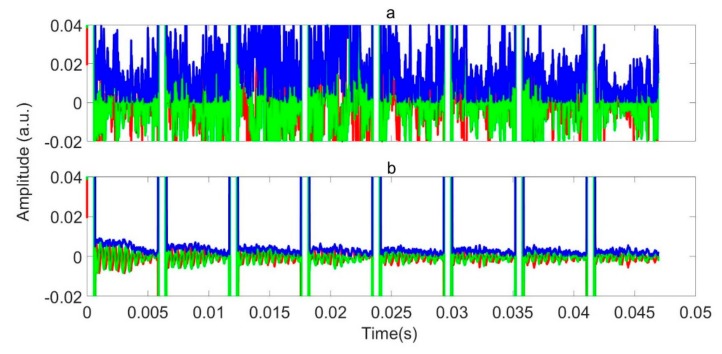
An averaged and demodulated signal. The red, green, and blue lines represent the real, imaginary, and magnitude of the data, respectively: (**a**) without and (**b**) with the application of the RFI decision tree identification model and removal algorithm.

The decision tree model for RFI identification and localization, followed by direct RFI removal and averaging, was applied to data series II and III. Here, we present a series of exemplars where a limited number of parameters were varied, in order to show the scale of improvement possible. Figure 12a shows the demodulated and averaged data for a set of 64 scans acquired at a frequency of 3.6034 MHz in the presence of RFI. The pulse length ($P_{90}$ = 223 μs) and the pulse gap is τ = 5868 μs. Only the train of $n_{echo}$ = 8 refocusing the pulses and echoes is shown repeating every  τ; the initial excitation pulse is excluded. The refocusing pulses appear as white vertical bands, where they saturate the receiver. More-or-less everywhere else, the data is completely corrupted by RFI, which, at any time point, may have occurred in just a handful of the averaged scans. However, no single scan within the average is completely RFI free.

Figure 12b shows the same data after the decision tree machine identification of RFI, demodulation, and RFI elimination by direct removal. The improvement is immediate and obvious. The oscillating quadrature components of the underlying NQR signal can be seen clearly. The oscillations reveal that the signal is off resonance by 2 kHz. This figure and analysis of a large number of experiments suggests that, as long as enough averages (data sets) are available, the direct removal is a satisfactory strategy to remove burst mode RFI once identified.

In order to evidence the impact of the different experimental parameters on obtaining a clean NQR signal in the presence of RFI, we showed the results of applying the algorithm to different experiments. In each case, without the new processing, the echo signals (where present) were completely obscured by RFI.

**Figure 13:**
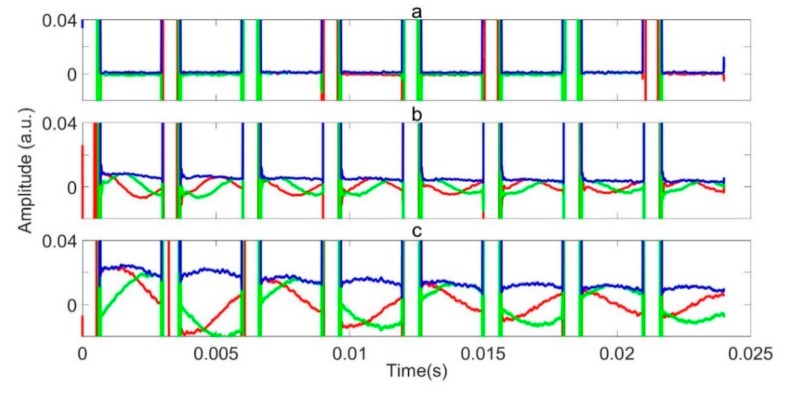
Application of the RFI suppression model on the data. The red, green, and blue lines represent the real, the imaginary, and the magnitude of the data, respectively, as follows: (**a**) of 0 g, (**b**) of 100 g, and (**c**) of 200 g. All of the other parameters are the same.

In Figure 13, we varied the sample mass, by 0, 100, and 200 g, but kept the other parameters constant and close to optimal, as follows: τ = 3000 µs, offset frequency $\delta f_{NQR}$ = 0.17 kHz, and $n_{echo}$ = 8 and $P_{90}$ = 240 µs. The observed signal amplitude was seen to scale with the sample mass. Importantly, no signal was seen with no sample.

In order to demonstrate the efficiency of the method while varying other key NQR parameters, namely, frequency, $P_{90}$, and τ, we showed three sets of processed data in Figure 13. In each case, the sample weight was 100 g, and the ideal parameters are about the following: $f_{\mathrm{NQR}}$ = 3.6051 MHz, $P_{90}=$ 240 µs, and τ = 3000 µs.

**Figure 14:**
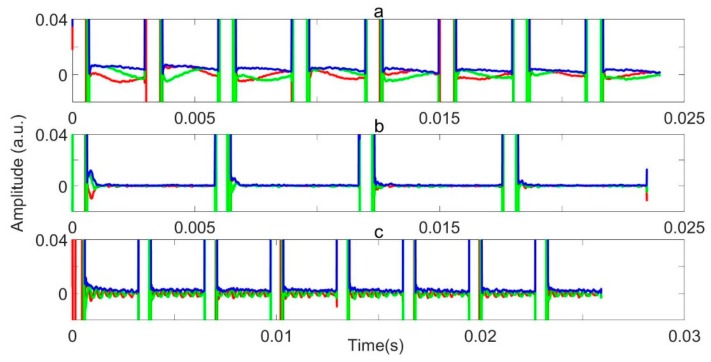
Application of RFI suppression model on data of the same 100 g weight sample, but varying key experimental parameters: see text for an explanation of the parameter changes in (**a**), (**b**) and (**c**). The red, green, and blue lines represent the real, imaginary, and magnitude of the data, respectively.

In Figure 14a, $\delta f_{\mathrm{NQR}}$ = 0.8 kHz, with $P_{90}\;=$ 240 µs and τ = 3000 µs. The signal can still be seen, but with slightly less amplitude. In a real security application, this scenario might result from an unusually warmed or cooled sample. In Figure 14b, the signal is not seen. This is because of a combination of things. In particular, the $P_{90}=$ 195 µs is sub optimum, which might result from a poorly calibrated instrument, and τ = 5866 µs is long. The offset $\delta f_{NQR}\;$= 0.3 kHz is probably not insignificant. In Figure 14c, $P_{90}=$ 200 µs, and τ = 3240 µs and $\delta f_{NQR}$ = 3.5 kHz. A small, but rapidly oscillating signal can be identified.

### 4.3. Choice of Decision Tree Model

In this work, we used the decision tree model. This is a well-established and robust machine learning protocol that is easily implemented. However, other alternatives are available, and have been used in other applications. Amongst these are K-nearest neighbors (KNN) [25] and support vector machine (SVM) [26]. We do not repeat the details here, suffice to say neither was as satisfactory as that of the decision tree for the RFI removal of NQR data. We used the same set of training data with the same *k* = 25-fold cross validation to compare with KNN and SVM. KNN gave about 95% accuracy and SVM gave about 91% accuracy compared to the decision tree model, which gave 98.2% accuracy for the correct identification of RFI.

## 5. Results of RFI Removal

A perceived security application involves human operators (in a later application, a machine recognition system) viewing a data set and making an instant go/no-go decision as to whether a signal has been detected.

In an attempt to mimic this, a trial was established where volunteers were first trained to recognize NQR signals in graphical plots of processed data sets, such as seen in Figure 13 and Figure 14. The volunteers were each then shown a random selection of 100 differently processed echo trains. A random, approximate half, of each 100 echo trains shown were demodulated and filtered in the normal way. The other half were treated for removal of RFI using the decision tree model, before identical demodulation and filtering. The data from series III was used for this exercise. The volunteers were asked to indicate their confidence, using a 0–10 scale, as follows: “NQR signal definitely present—10”, indicating contraband present, down to “NQR signal definitely not present—0”, meaning no contraband present. A score of five meant that they were uncertain and could not decide if a signal was, or was not, present.

**Figure 15:**
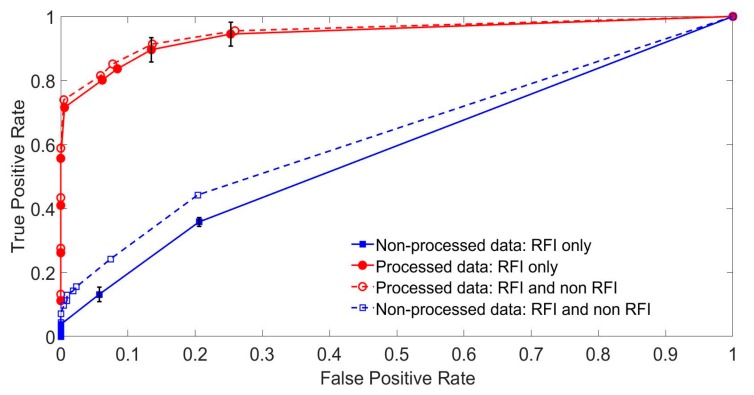
Receiver operating characteristic (ROC) curves comparing the results of voting for signals appearance, which are processed by the proposed algorithm (red curve), and not processed (blue curve). The dashed lines represent the results when all signals contain RFI, and the solid lines represent a mixture or RFI and non RFI.

Receiver operating characteristic (ROC) curves are a standard tool to analyze the results of such a trial [20]. They are routinely used to illustrate the efficiency of RFI removal schemes from NQR data, although not, as far as we are aware, for using blind trials with human volunteers inspecting the data, as we used here (see e.g., [9,10,11,12,13,14,15]). They measure the rate at which a model correctly predicts a true positive result for a given rate of false positive (or false alarm) identification. For the ROC analysis presented here, the volunteer scores were separated post trial, back into those arising from the RFI decision tree treated and not-treated data. Furthermore, the volunteer score ratings were converted (linear scaling) to a probability likelihood (0 to 1). A decision threshold was set from which the true and false positive identification rates could be calculated as a function of the decision threshold. The data from the 21 volunteers were combined. In this way, the improvement brought by the RFI identification and removal could be quantified. The exercise yielded two roughly equal fractions of data, namely: with and without decision tree identification, and the direct removal of RFI. Based on the aggregated volunteer scores arising from the human trial, two receiver operating characteristic (ROC) curves were calculated for each fraction. The curves are shown is Figure 15. In this figure, the thick red line is for the data containing an RFI that has been processed with RFI removal, as well as demodulation and filtering, whereas the thick blue line is for demodulation and filtering only.

The respective areas under curve (AUC) are 0.906 and 0.580, strongly endorsing two observations. First, without RFI removal, the data is almost useless as “guessing” yields using the diagonal line with AUC = 0.5. Second, RFI removal has a very positive impact. Under ideal scenarios, the “curve” is square and AUC = 1.

In order to test whether active RFI suppression negatively affects signal detection when there is no RFI present (i.e., does RFI suppression make the result worse when no RFI is present due to miss-classification of data units?), we further calculated and added two more ROC curves, these for the data sets that specifically included data when no RFI was present. These are shown as the dotted lines in Figure 15; as these two curves are very similar to before, we conclude that when the RFI is not present in the data, the algorithm does not overly classify the NQR echo signal as RFI and make matters worse.

## 6. Conclusions

We have shown that a decision tree model, machine learning algorithm is very well suited to the problem of identifying short radio frequency interference bursts, as they may corrupt the NQR signals enabling strategies to suppress these burst from the measured signals. Burst mode RFI, for instance, because of air craft communications, is not the only source of RFI at low MHz frequencies that may corrupt NQR signals, but, in the environs of our laboratory, it is by far the most dominant. It is less likely that this method could be successfully used on a lower level, continuous background RFI, although there is clear opportunity to develop the methodology further by incorporating the additional data available from the other RFI sensors—this is the subject of ongoing work.

Once RFI is detected, a strategy is required to suppress it. Here, we have used a simple method of direct removal, and averaging the remaining data across multiple scans. This strategy is effective here for the RFI incidence rate studied, and with 64 scans available to average. However, with this strategy, care must be taken to ensure that sufficient averages are recorded, so as to ensure that at least one data set is contributed at all times. Notwithstanding that 64 scans were available per data set, in practice, only two or three good units were available to contribute to part of an echo in some cases.

A possible alternate strategy for RFI removal post identification is linear predictive coding. We have previously explored this in the related context of RFI corrupting very short duration echoes. The RFI was predicted through the echo, and was subtracted out. The reverse scenario is used to predict the long echo through the burst of RFI. It may have benefit when only a very few (<64) averages are available. It would also be interesting to test a boosted decision tree methodology, and to compare the enhancement in term of the accuracy and speed of the model.

The ROC curves shown here do not discriminate the data as a function of the sample mass, and hence, sensitivity is not assessed. Such an analysis is very much application specific.
